# Effect of esketamine on postoperative pain relief and depressive status in patients with traumatic fractures

**DOI:** 10.3389/fmed.2025.1684134

**Published:** 2025-11-12

**Authors:** Zhongyu Liu, Jinhui Xu, Mingsheng Zhang, Tao Zhou

**Affiliations:** Department of Anesthesiology, Jiangxi Provincial People's Hospital, The First Affiliated Hospital of Nanchang Medical College, Nanchang, Jiangxi, China

**Keywords:** esketamine, traumatic fractures, postoperative analgesia, depression, anesthesiology

## Abstract

**Objective:**

To investigate the effects of different doses of esketamine combined with sufentanil on postoperative pain relief and depressive states in patients with traumatic fractures.

**Methods:**

This prospective, randomized, triple-blind, placebo-controlled trial (registered at the Chinese Clinical Trial Registry, Identifier: ChiCTR2100054238) enrolled 225 patients with traumatic lower limb fractures (ASA I-III, aged 18–64) at Jiangxi Provincial People's Hospital between September 2021 and June 2024. Patients were randomly allocated to three groups (*n* = 75 each). All received a standard postoperative analgesic pump (sufentanil 2 μg/kg + tropisetron 10 mg in 100 ml saline, 1.5 ml/h basal rate, 2 ml PCA bolus, 20 min lockout). Concurrently, they received a 24-h continuous infusion via a separate pump: Group L (low-dose) received esketamine 0.5 mg/kg in 48 ml saline (2 ml/h); Group H (high-dose) received esketamine 0.75 mg/kg in 48 ml saline (2 ml/h); Group C (control) received 48 ml saline (2 ml/h). Primary outcomes were Hamilton Depression Rating Scale (HAMD) scores (assessed preoperatively, and on postoperative days 1, 3, 7) and total sufentanil consumption. Secondary outcomes included Visual Analog Scale (VAS) pain scores, PCA compressions, serum BDNF/IL-6 levels, and adverse events.

**Results:**

Both esketamine groups (L and H) demonstrated significantly lower HAMD scores on postoperative days 1 and 3 compared to group C (all *P* < 0.05), with group H showing a greater reduction than group L on day 3 (*P* < 0.05). Postoperative sufentanil consumption and PCA compressions were significantly reduced in groups L and H vs. C (*P* < 0.05). Group H also had significantly fewer PCA compressions than group L (*P* < 0.05). The VAS score was significantly lower in group H than in group C at 12 h post-surgery (*P* < 0.05). The incidence of postoperative nausea/vomiting was significantly lower in groups L and H compared to group C (*P* < 0.05).

**Conclusion:**

Continuous postoperative infusion of esketamine (0.5–0.75 mg/kg over 24 h) in patients with traumatic lower limb fractures effectively alleviates postoperative depressive symptoms, provides opioid-sparing analgesia, reduces opioid-related adverse effects like nausea and vomiting, and is associated with increased BDNF and decreased IL-6 levels.

## 1 Introduction

Traumatic fractures are a prevalent type of orthopedic condition. Globally, the burden of fractures remains substantial, with an estimated 178 million new cases in 2019, representing a 33.4% increase since 1990, largely driven by population growth and aging (1). Traumatic fractures often trigger acute stress responses in patients. These individuals not only experience significant physiological changes due to severe physical damage but are also prone to psychological disturbances, such as anxiety, depression, and irritability (2). These negative emotional states are major factors contributing to poor postoperative recovery or prolonged recovery times (3, 4). Furthermore, a critical and often under-addressed aspect is the intricate, bidirectional relationship between postoperative pain and psychological distress. Acute postoperative pain can exacerbate depressive symptoms, while pre-existing anxiety and depression can, in turn, lower pain thresholds and increase the risk of developing chronic pain, creating a vicious cycle that impedes recovery (5). Factors such as pain catastrophizing—a negative cognitive and emotional response to pain—have been identified as significant predictors of increased postoperative opioid consumption and poorer functional outcomes (6). This highlights the necessity of a holistic treatment approach that simultaneously targets both pain and its psychological comorbidities.

As an essential component of multimodal analgesia, N-methyl-D-aspartate (NMDA) receptor antagonists are widely used in clinical practice. Ketamine, a representative drug of this class, has been shown in previous studies to rapidly improve depressive states in patients at subclinical doses (7). Esketamine, the S(+) enantiomer of ketamine, exhibits twice the affinity for NMDA receptors compared to ketamine, while significantly reducing cardiovascular and psychiatric side effects (8). This dual-action potential makes esketamine a particularly compelling agent for the trauma population, as it offers the promise of concurrently managing acute postoperative pain while mitigating the concurrent depressive symptoms that are highly prevalent in these patients. However, the use of esketamine is not without controversy; concerns regarding psychomimetic side effects remain, necessitating careful dose titration and patient monitoring (9, 10). Furthermore, a standardized dosing regimen for esketamine in the context of postoperative analgesia has not been established. While studies on depression have utilized various doses, its optimal dose for simultaneously managing post-traumatic pain and depressive symptoms remains to be determined. This study explored the effects of different doses of esketamine combined with sufentanil on postoperative analgesia and depressive states in patients with traumatic fractures. The aim was to provide a theoretical basis for optimizing anesthesia, improving multimodal postoperative analgesia, and accelerating recovery in trauma orthopedic patients.

## 2 Materials and methods

### 2.1 General information

Patients with traumatic fractures who underwent orthopedic surgery at Jiangxi Provincial People's Hospital from September 2021 to June 2024 were enrolled in the study. The inclusion criteria were: (1) lower limb fracture; (2) age 18–64 years old; (3) American Society of Anesthesiologists (ASA) grade I-III; (4) Hamilton depression rating scale (HAMD) 17-item score ≥ 7 upon admission; (5) no prior diagnosis of major depressive disorder or other mental illnesses; (6) body mass index (BMI) between 18–35 kg/m^2^. Exclusion criteria were: (1) allergies to esketamine; (2) patients who did not agree to be included in the study; (3) patients with refractory, intractable hypertension and severe cardiovascular and cerebrovascular diseases; (4) patients with glaucoma; (5) patients with sleep apnea syndrome; (6) those who could not complete the questionnaire. The estimated sample size was calculated using PASS software version 15.0 (NCSS). Based on our preliminary experimental results [which indicated that the mean ± standard deviation of the HAMD score on postoperative day 3 was 13.5 ± 4.2 in the control group and 10.5 ± 5.8 in a group receiving esketamine, with an effect size (f) of 0.32 calculated from these means and pooled standard deviation], a two-tailed α = 0.05 was used, and the test power (1-β) = 0.9. The sample size calculation method for multiple sample mean comparisons in a completely randomized design was selected, with a dropout rate of 20% was assumed. This accounted for potential patient dropouts due to reasons such as withdrawal of consent, protocol deviation, or loss to follow-up. Finally, 225 patients who met the criteria were included and randomly assigned in a 1:1:1 ratio to a low-dose group (L), a high-dose group (H), or a control group (C), with 75 people in each group. The randomization sequence was generated using computer software (IBM SPSS Statistics, Version 22.0) by an independent statistician not involved in the trial. The group assignment was concealed in sequentially numbered, opaque, sealed envelopes, which were opened by an anesthesia nurse not involved in the study after patient enrollment. This study was approved by the Medical Ethics Committee of Jiangxi Provincial People's Hospital (Approval No.: 2021-023 IIT). Informed consent was obtained from all participants. A triple-blind design was used. The anesthesiologists performing the procedures, the participants, and the follow-up staff were blinded to group allocation. Both the analgesic pumps and esketamine pump labels were identical to ensure blinding.

### 2.2 Research methods

General anesthesia was administered. The induction agents included midazolam, rocuronium bromide, sufentanil, and etomidate. Anesthesia maintenance was achieved with sevoflurane inhalation combined with continuous infusion of propofol and remifentanil, with intermittent boluses of rocuronium bromide. Intraoperative parameters were maintained as follows: PaCO2 35–45 mmHg, MAP ≥ 65 mmHg, and BIS between 45–55. Postoperatively, all patients received ropivacaine hydrochloride infiltration at the surgical site for local anesthesia. The postoperative analgesia pump formula consisted of sufentanil (2 μg/kg) and tropisetron (10 mg), diluted with normal saline to a total volume of 100 ml, and the pump speed was 1.5 ml/h for continuous infusion. The patient-controlled analgesia (PCA) dose was 2 ml/activation, the maximum dose did not exceed 10 ml/h, and the lockout interval was 20 min, and the PCA lasted for 48 h after surgery (additional dosage was administered if analgesic drugs were depleted within 48 h). The formula of esketamine pump was as follows: Group L, esketamine hydrochloride injection (Jiangsu Hengrui Medicine Co., Ltd., 2 ml/50 mg, batch number 230520BL) at 0.5 mg/kg, diluted with saline to 48 ml, infused at 2ml/h for 24h. Group H, esketamine hydrochloride injection at 0.75 mg/kg, diluted with saline to 48 ml, infused at 2 ml/h for 24 h. Group C, saline (48 ml) infused at 2 ml/h for 24 h. Both the esketamine and analgesic pumps were initiated immediately after surgery. The esketamine pump was discontinued after 24 h, while the analgesic pump was discontinued after 48 h. If the patient had nausea and vomiting during the use of the pump, azasetron injection was administered as needed, and midazolam was used as needed to treat psychiatric symptoms.

### 2.3 Observation indicators

Baseline demographic and clinical characteristics, including age, gender, weight, body mass index (BMI), American Society of Anesthesiologists (ASA) physical status classification, and the presence of comorbidities (hypertension, diabetes mellitus), were recorded upon enrollment. Intraoperative data, including the type of fracture and surgery, duration of surgery and anesthesia, total intraoperative sufentanil consumption, and estimated blood loss, were also collected to assess the homogeneity across study groups.

The primary outcomes of this study were the HAMD score and the total postoperative sufentanil consumption. The HAMD scores were assessed before surgery and on postoperative days 1, 3, and 7. The total postoperative sufentanil dosage was recorded at the conclusion of the 48-h analgesic pump infusion period.

The secondary outcomes encompassed pain intensity, sedation level, analgesic demand, serum biomarkers, and the incidence of adverse reactions. Pain intensity was measured using the Visual Analog Scale (VAS) score, and the level of sedation was assessed using the Ramsay sedation score. These assessments, along with vital signs monitoring including blood pressure, heart rate, respiratory rate, and blood oxygen saturation, were conducted at specific time points during the use of the postoperative analgesia pump: 4 h (T1), 8 h (T2), 12 h (T3), 24 h (T4), and 48 h (T5) after surgery. The number of PCA compressions was recorded from the pump's internal history as a measure of analgesic demand. Serum levels of Brain-Derived Neurotrophic Factor (BDNF) and Interleukin-6 (IL-6) were analyzed from fasting venous blood samples collected before surgery, and at 24 and 48 h after surgery. Serum concentrations of BDNF and IL-6 were quantified using commercial enzyme-linked immunosorbent assay (ELISA) kits (Human BDNF ELISA Kit, RayBiotech, catalog # ELH-BDNF-1; Human IL-6 ELISA Kit, Invitrogen, catalog # KHC0061). All assays were performed strictly according to the manufacturers' instructions. The minimum detectable doses (sensitivity) for the assays were < 20 pg/mL for BDNF and < 2 pg/mL for IL-6. The intra-assay coefficients of variation were < 8% for both assays, and the inter-assay coefficients of variation were < 12% for both assays, as validated in our laboratory prior to the study commencement. All samples were measured in duplicate. Adverse reactions, including hypotension, hypertension, hypoxemia, nausea, vomiting, dizziness, nightmares, and delirium, were meticulously recorded throughout the postoperative observation period.

### 2.4 Statistical methods

Data analysis was performed following the intention-to-treat (ITT) principle, wherein all randomized patients were analyzed in the groups to which they were originally assigned. Patients who dropped out were included in the analysis using the last observation carried forward (LOCF) method for the HAMD score data. SPSS 22.0 (IBM Corp., Armonk, NY, USA) was used for data analysis. Normally distributed quantitative data were expressed as mean ± standard deviation. For outcomes measured repeatedly over time (e.g., HAMD scores, VAS scores, Ramsay scores, BDNF, and IL-6 levels), two-way repeated-measures analysis of variance (ANOVA) was employed to examine the main effects of Group and Time, as well as their interaction. If the interaction effect was statistically significant, *post-hoc* analyses were conducted using one-way ANOVA with Bonferroni correction for multiple comparisons at each time point, and pairwise comparisons between groups were reported as mean differences with 95% confidence intervals (CIs). For continuous outcomes measured at a single time point (e.g., postoperative sufentanil dosage, PCA compressions), one-way ANOVA was used for group comparisons, followed by Bonferroni *post-hoc* tests with mean differences and 95% CIs. Categorical data were expressed as *n* (%) and compared using the χ2 test or Fisher's exact test, as appropriate. Relative risk (RR) with 95% CIs was calculated for adverse events. Statistical significance was set at *P* < 0.05.

## 3 Results

### 3.1 Comparison of baseline and surgical characteristics

There were no statistically significant differences in baseline demographic and clinical characteristics (including age, gender, weight, body mass index, ASA physical status classification, and the prevalence of comorbidities) or in surgical conditions (including fracture type, surgery type, duration of surgery, anesthesia duration, total intraoperative sufentanil consumption, and estimated blood loss) among the three groups (all *P* > 0.05, Tables 1, 2).

**Table 1:** Comparison of baseline characteristics among the three groups.

Age (years)	45.9 ± 8.7	47.2 ± 7.9	46.2 ± 9.3	0.652
Gender (M/F)	37/38	43/32	29/46	0.108
Weight (kg)	58.2 ± 13.3	55.9 ± 12.6	61.5 ± 14.5	0.054
BMI (kg/m^2^)	23.1 ± 3.2	22.8 ± 3.0	23.4 ± 3.5	0.487
ASA Grade (I/II/III)	12/48/15	10/50/15	11/49/15	0.945
Comorbidities (*n*, %)	8 (10.7%)	7 (9.3%)	9 (12.0%)	0.874
Age (years)	45.9 ± 8.7	47.2 ± 7.9	46.2 ± 9.3	0.652

Data are presented as mean ± standard deviation or number. ASA, American Society of Anesthesiologists. Comorbidities include hypertension and/or diabetes mellitus.

**Table 2:** Comparison of surgical and intraoperative characteristics among the three groups.

**Characteristic**	**C Group (*n =* 75)**	**L Group (*n =* 75)**	**L Group (*n =* 75)**	***P* Value**
**Fracture type**, ***n*** **(%)**				
Femur	40 (53.3%)	43 (57.3%)	38 (50.7%)	0.723
Tibia/Fibula	35 (46.7%)	32 (42.7%)	37 (49.3%)	
**Surgery type**, ***n*** **(%)**				
Intramedullary nail	43 (57.3%)	41 (54.7%)	42 (56.0%)	0.956
Plate	32 (42.7%)	34 (45.3%)	33 (44.0%)	
Duration of surgery (min)	126.1 ± 32.6	135.2 ± 39.3	131.5 ± 27.3	0.289
Anesthesia duration (min)	145.3 ± 25.1	152.7 ± 30.8	148.9 ± 28.5	0.312
Intraoperative sufentanil (μg)	25.8 ± 4.5	26.5 ± 5.1	25.2 ± 4.8	0.215
Blood loss (ml)	312.3 ± 41.2	378.2 ± 39.7	349.6 ± 36.7	0.089

Data are presented as mean ± standard deviation or number (percentage). No significant differences were found among groups for any variable.

### 3.2 Comparison of HAMD scores over time

**Figure 1:**
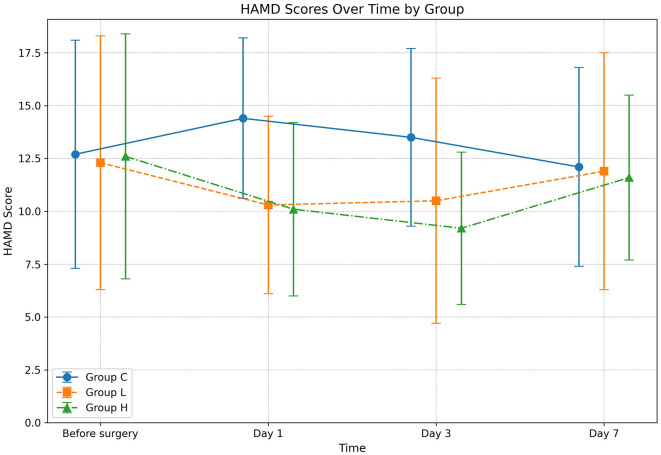
Changes in Hamilton Depression Rating Scale (HAMD) scores over time. The graph displays the mean HAMD scores for Group C (solid line), Group L (dashed line), and Group H (dash-dot line) at four distinct time points: before surgery, and on days 1, 3, and 7 post-surgery. Data are presented as mean ± standard deviation, with error bars indicating the standard deviation at each time point. Statistical analysis was performed using a two-way repeated-measures ANOVA.

The omnibus two-way repeated-measures ANOVA revealed significant main effects of Group (*P* < 0.001) and Time (*P* = 0.012), as well as a significant Group × Time interaction (*P* = 0.003) on HAMD scores (Table 3). *Post-hoc* analyses demonstrated that compared with group C, the HAMD scores of groups L and H were significantly lower on days 1 and 3 after surgery (all *P* < 0.001); compared with group L, the HAMD score of group H was significantly lower on day 3 after surgery (Mean Difference −1.3, 95% CI [−2.8, −0.2], *P* = 0.022). No significant between-group differences were observed at day 7 (all *P* > 0.05) (Table 4; Figure 1).

**Table 3:** Comparison of HAMD scores among the three groups at different time points.

**Group**	**N**	**Before surgery**	**Day 1**	**Day 3**	**Day 7**	***P* value (Group)**	***P* value (Time)**	***P* value (Group × Time)**
C	75	12.7 ± 5.4	14.4 ± 3.8	13.5 ± 4.2	12.1 ± 4.7	< 0.001	0.012	0.003
L	75	12.3 ± 6.0	10.3 ± 4.2^a^	10.5 ± 5.8^a^	11.9 ± 5.6			
H	75	12.6 ± 5.8	10.1 ± 4.1^a^	9.2 ± 3.6^ab^	11.6 ± 3.9			

Data are presented as mean ± standard deviation. The omnibus test was performed using two-way repeated-measures ANOVA to examine the effects of Group, Time, and their Interaction. The between-group comparisons at each time point were conducted using one-way ANOVA with Bonferroni correction. CI, Confidence Interval. ^a^*P* < 0.05 compared with group C; ^b^*P* < 0.05 compared with group L (from omnibus ANOVA with *post-hoc* tests).

**Table 4:** *Post-hoc* comparisons of HAMD scores between groups at each postoperative time point.

**Time point**	**Comparison**	**Mean difference**	**95% CI**	***P* value**
Day 1	L vs. C	−4.1	[−5.3, −2.9]	< 0.001
	H vs. C	−4.3	[−5.5, −3.1]	< 0.001
	H vs. L	−0.2	[−1.4, 1.0]	0.743
Day 3	L vs. C	−3.0	[−4.5, −1.5]	< 0.001
	H vs. C	−4.3	[−5.8, −2.8]	< 0.001
	H vs. L	−1.3	[−2.8, 0.2]	0.022
Day 7	L vs. C	−0.2	[−1.7, 1.3]	0.796
	H vs. C	−0.5	[−2.0, 1.0]	0.514
	H vs. L	−0.3	[−1.8, 1.2]	0.691

Data are presented as Mean Difference and 95% Confidence Interval (CI). *Post-hoc* comparisons were performed only if the omnibus Group × Time interaction was significant. *P* values are adjusted for multiple comparisons using the Bonferroni method.

### 3.3 Comparison of VAS and Ramsay scores over time

**Figure 2:**
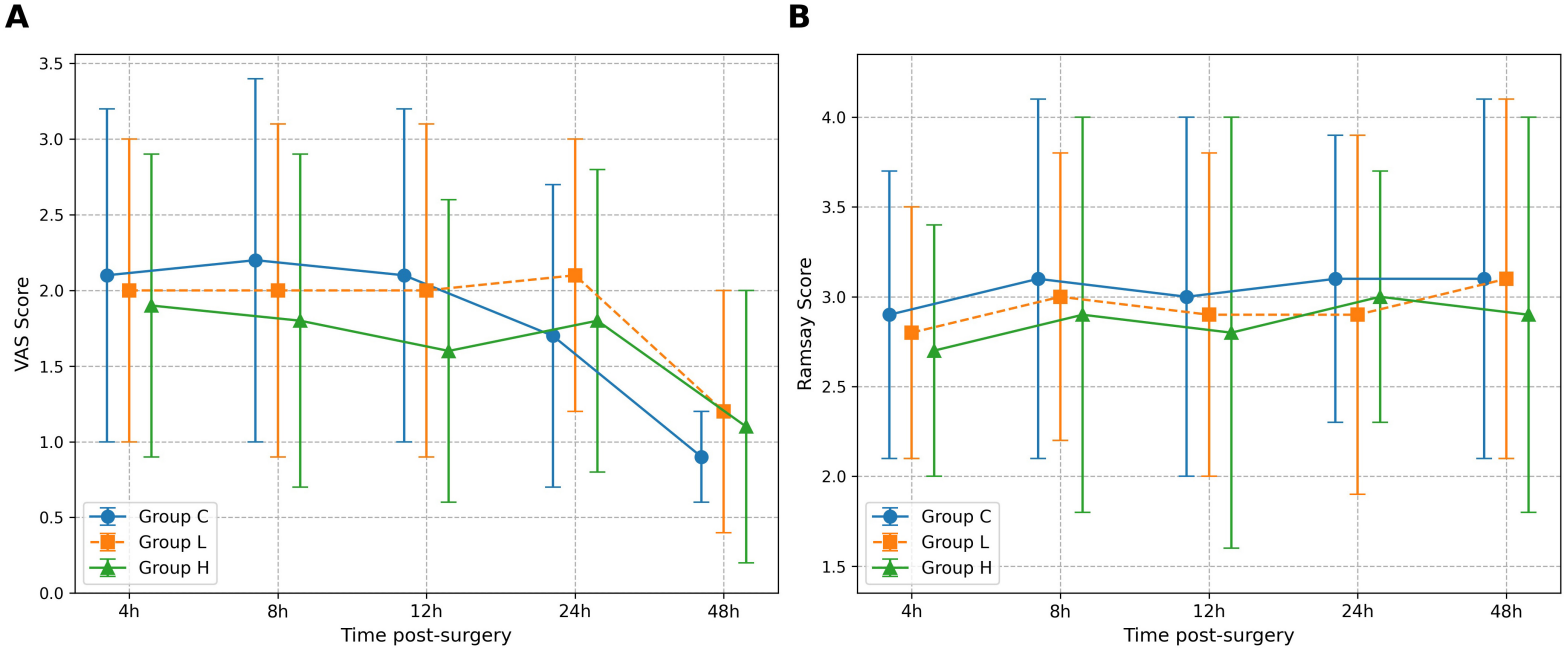
Comparison of Visual Analog Scale (VAS) and Ramsay sedation scores over time. The figure illustrates the postoperative scores for the three study groups: Group C (solid line), Group L (dashed line), and Group H (dash-dot line). **(A)** The graph displays the mean VAS scores for pain. **(B)** The graph shows the mean Ramsay sedation scores. Data are presented as mean ± standard deviation, with error bars representing the standard deviation. Measurements were taken at five post-surgery time points: 4 h (T1), 8 h (T2), 12 h (T3), 24 h (T4), and 48 h (T5). A two-way repeated-measures ANOVA was used to analyze the effects of group, time, and their interaction.

For VAS scores, the omnibus test showed significant main effects of Group (*P* = 0.035) and Time (*P* < 0.001), but no significant Group × Time interaction (*P* = 0.128) (Table 5). *Post-hoc* comparisons at individual time points revealed that only at T3 (12 h post-surgery) did group H show significantly lower VAS scores compared to group C (Mean Difference −0.5, 95% CI [−0.9, −0.1], *P* = 0.021) (Table 6). No other significant between-group differences were found at other time points. For Ramsay sedation scores, no significant main effects or interaction were observed (all *P* > 0.05) (Table 5; Figure 2).

**Table 5:** Comparison of VAS and Ramsay scores at different time points in the three groups.

**Score**	**Group**	**N**	**T1 (4 h)**	**T2 (8 h)**	**T3 (12 h)**	**T4 (24 h)**	**T5 (48 h)**	***P* value (Group)**	***P* value (Time)**	***P* value (Group × Time)**
VAS	C	75	2.1 ± 1.1	2.2 ± 1.2	2.1 ± 1.1	1.7 ± 1.0	0.9 ± 0.3	0.035	< 0.001	0.128
	L	75	2.0 ± 1.0	2.0 ± 1.1	2.0 ± 1.1	2.1 ± 0.9	1.2 ± 0.8			
	H	75	1.9 ± 1.0	1.8 ± 1.1	1.6 ± 1.0^a^	1.8 ± 1.0	1.1 ± 0.9			
Ramsay	C	75	2.9 ± 0.8	3.1 ± 1.0	3.0 ± 1.0	3.1 ± 0.8	3.1 ± 1.0	0.421	0.087	0.754
	L	75	2.8 ± 0.7	3.0 ± 0.8	2.9 ± 0.9	2.9 ± 1.0	3.1 ± 1.0			
	H	75	2.7 ± 0.7	2.9 ± 1.1	2.8 ± 1.2	3.0 ± 0.7	2.9 ± 1.1			

Data are presented as mean ± standard deviation. The omnibus test was performed using two-way repeated-measures ANOVA to examine the effects of Group, Time, and their Interaction. T1: 4 h; T2: 8 h; T3: 12 h; T4: 24 h; T5: 48 h post-surgery. ^a^*P* < 0.05 compared with group C.

**Table 6:** *Post-hoc* comparisons of VAS scores between groups at significant time points.

**Time point**	**Comparison**	**Mean Difference**	**95% CI**	***P* value**
T3 (12 h)	H vs. C	−0.5	[−0.9, −0.1]	0.021
	L vs. C	−0.1	[−0.5, 0.3]	0.593
	H vs. L	−0.4	[−0.8, 0.0]	0.051

Data are presented as Mean Difference and 95% Confidence Interval (CI). *Post-hoc* comparisons were performed only for time points where the overall group effect or interaction was significant. *P* values are adjusted for multiple comparisons using the Bonferroni method. Only significant or relevant comparisons are detailed.

### 3.4 Postoperative analgesic consumption

Both esketamine groups significantly reduced postoperative sufentanil consumption compared to group C (L vs. C: MD −13.8 μg, 95% CI [−15.3, −12.3], *P* < 0.001; H vs. C: MD −16.3 μg, 95% CI [−17.8, −14.8], *P* < 0.001) (Table 7). Group H also consumed significantly less sufentanil than group L (MD −2.5 μg, 95% CI [−4.0, −1.0], *P* = 0.001) (Table 8). Similarly, PCA compressions were significantly reduced in both esketamine groups vs. control (L vs. C: MD −6.2, 95% CI [−7.2, −5.2], *P* < 0.001; H vs. C: MD −9.4, 95% CI [−10.2, −8.6], *P* < 0.001), with group H showing significantly fewer compressions than group L (MD −3.2, 95% CI [−3.8, −2.6], *P* < 0.001) (Tables 7, 8).

**Table 7:** Postoperative sufentanil consumption and PCA compressions with effect sizes vs. control group.

**Group**	**N**	**Sufentanil dosage (μg)**	**Mean Difference [95% CI] vs. C**	***P* value vs. C**	**PCA compressions**	**Mean Difference [95% CI] vs. C**	***P* value vs. C**
C	75	83.0 ± 5.6	Reference	-	11.5 ± 3.5	Reference	-
L	75	69.2 ± 4.8	−13.8 [−15.3, −12.3]	< 0.001	5.3 ± 2.8	−6.2 [−7.2, −5.2]	< 0.001
H	75	66.7 ± 5.1	−16.3 [−17.8, −14.8]	< 0.001	2.1 ± 1.2	−9.4 [−10.2, −8.6]	< 0.001

Data are presented as mean ± standard deviation. MD, Mean Difference; CI, Confidence Interval. Group comparisons for each variable were performed using one-way ANOVA. The *P* value vs. C column represents the result of *post-hoc* testing (Bonferroni) comparing each esketamine group to the Control group.

**Table 8:** Pairwise comparisons between esketamine groups for postoperative analgesic consumption.

**Comparison**	**Sufentanil MD [95% CI]**	***P* value**	**PCA MD [95% CI]**	***P* value**
H vs. L	−2.5 [−4.0, −1.0]	0.001	−3.2 [−3.8, −2.6]	< 0.001

Data are presented as Mean Difference and 95% Confidence Interval (CI). The comparison between the high-dose (H) and low-dose (L) esketamine groups was performed using an independent samples *t*-test with Bonferroni correction.

### 3.5 Incidence of adverse reactions

Compared with group C, the incidence of postoperative nausea and vomiting was significantly reduced in both group L and group H (RR 0.30, 95% CI [0.09, 1.02], *P* < 0.05 for both) (Table 9). No statistically significant differences were observed among the three groups in other adverse reactions including hypotension, hypertension, hypoxemia, dizziness, nightmares, or delirium (all *P* > 0.05).

**Table 9:** Incidence of adverse reactions after surgery in three groups.

**Group**	**N**	**Hypotension**	**Hypertension**	**Hypoxemia**	**Nausea/ Vomiting**	**RR [95% CI] vs. C**	**Dizziness**	**Nightmare**	**Delirium**
C	75	3 (4.0%)	5 (6.7%)	0 (0.0%)	10 (13.3%)	Reference	7 (9.3%)	0 (0.0%)	0 (0.0%)
L	75	0 (0.0%)	5 (6.7%)	0 (0.0%)	3 (4.0%)^a^	0.30 [0.09, 1.02]	10 (13.3%)	2 (2.7%)	0 (0.0%)
H	75	2 (2.7%)	3 (4.0%)	0 (0.0%)	3 (4.0%)^a^	0.30 [0.09, 1.02]	10 (13.3%)	2 (2.7%)	0 (0.0%)

Data are presented as number (percentage). RR, Relative Risk; CI, Confidence Interval. ^a^*P* < 0.05 compared with group C by Chi-square test with Bonferroni correction. Only nausea/vomiting showed statistically significant differences between groups.

### 3.6 BDNF and IL-6 levels

**Figure 3:**
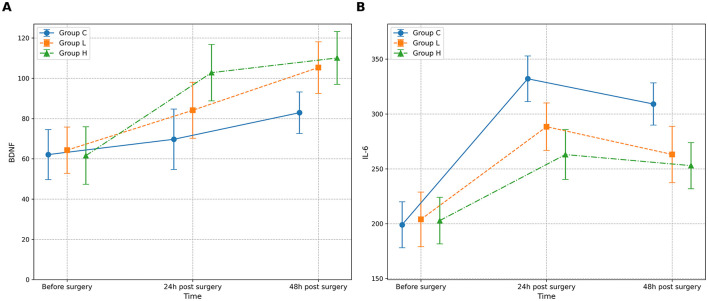
Changes in serum biomarker levels across groups over time. The figure displays the mean concentrations of **(A)** Brain-Derived Neurotrophic Factor (BDNF) and **(B)** Interleukin-6 (IL-6) for the three study groups: Group C (solid line), Group L (dashed line), and Group H (dash-dot line). Measurements were performed before surgery, and at 24 and 48 h post-surgery. Data are presented as mean ± standard deviation, with error bars indicating the standard deviation. A two-way repeated-measures ANOVA was used for statistical analysis.

The omnibus two-way repeated-measures ANOVA revealed significant main effects of Group and Time, as well as significant Group × Time interactions for both BDNF and IL-6 levels (all *P* < 0.001) (Table 10; Figure 3). *Post-hoc* analyses showed that compared with group C, BDNF levels in groups L and H were significantly increased at 24 and 48 h after surgery (all *P* < 0.001), while IL-6 levels were significantly decreased (all *P* < 0.001) (Table 11). Compared with group L, the BDNF level in group H was significantly higher at 24 h after surgery (MD 18.7, 95% CI [14.1, 23.3], *P* < 0.001), and the IL-6 level was significantly lower (MD −25.4, 95% CI [−32.5, −18.3], *P* < 0.001) (Table 11).

**Table 10:** Results of two-way repeated measures ANOVA for BDNF and IL-6 levels across groups over time.

**Index**	**Group**	**N**	**Before surgery**	**24 h post surgery**	**48 h post surgery**	***P* value (Group)**	***P* value (Time)**	***P* value (Group × Time)**
BDNF	C	75	62.1 ± 12.4	69.7 ± 15.0	82.9 ± 10.3	< 0.001	< 0.001	< 0.001
	L	75	64.3 ± 11.5	84.1 ± 13.9^a^	105.3 ± 12.8^a^						
	H	75	61.6 ± 14.3	102.8 ± 14.0^ab^	110.1 ± 13.1^a^						
IL-6	C	75	199.0 ± 21.0	332.1 ± 20.8	309.1 ± 19.3	< 0.001	< 0.001	< 0.001
	L	75	203.9 ± 24.9	288.4 ± 21.6^a^	263.1 ± 25.7^a^						
	H	75	202.8 ± 21.2	263.0 ± 22.7^ab^	252.9 ± 21.1^a^						

Data are presented as mean ± standard deviation. The omnibus test was performed using two-way repeated-measures ANOVA to examine the effects of Group, Time, and their Interaction. BDNF, Brain-Derived Neurotrophic Factor; IL-6, Interleukin-6. ^a^*P* < 0.05 compared with group C; ^b^*P* < 0.05 compared with group L (from omnibus ANOVA with *post-hoc* tests).

**Table 11:** *Post-hoc* comparisons of BDNF and IL-6 levels between groups at postoperative time points.

**Index**	**Time point**	**Comparison**	**Mean difference**	**95% CI**	***P* value**
BDNF	24 h	L vs. C	14.4	[9.8, 19.0]	< 0.001
		H vs. C	33.1	[28.5, 37.7]	< 0.001
		H vs. L	18.7	[14.1, 23.3]	< 0.001
	48 h	L vs. C	22.4	[18.3, 26.5]	< 0.001
		H vs. C	27.2	[23.1, 31.3]	< 0.001
		H vs. L	4.8	[0.7, 8.9]	0.021
IL-6	24 h	L vs. C	−43.7	[−50.8, −36.6]	< 0.001
		H vs. C	−69.1	[−76.2, −62.0]	< 0.001
		H vs. L	−25.4	[−32.5, −18.3]	< 0.001
	48h	L vs. C	−46.0	[−53.6, −38.4]	< 0.001
		H vs. C	−56.2	[−63.8, −48.6]	< 0.001
		H vs. L	−10.2	[−17.8, −2.6]	0.009

Data are presented as Mean Difference and 95% Confidence Interval (CI). *Post-hoc* comparisons were conducted following a significant omnibus Group × Time interaction. All pairwise comparisons at 24 h and 48 h post-surgery are shown. *P* values are adjusted for multiple comparisons using the Bonferroni method.

## 4 Discussion

Traumatic fractures are often caused by accidental events and require prolonged recovery, significantly affecting patients' daily lives. This leads to a higher likelihood of negative emotions (11). Studies have reported that the probability of acute stress reactions in patients with traumatic fractures ranges from 18.5% to 43.5% (12). Additionally, the incidence of depressive symptoms 1 month after trauma treatment reaches 20%, increasing to as high as 56% after 5–8 months (13). In the preliminary phase of this study, a total of 351 traumatic fracture patients requiring surgical treatment were evaluated. Preoperative HAMD scoring revealed that 125 patients (35.61%) had a score ≥7, indicating preoperative depressive states. Previous research has confirmed a strong negative correlation between musculoskeletal function recovery and anxiety or depression scores in patients with traumatic fractures (14). However, in current clinical practice, patients' negative emotions are often overlooked, with no proactive interventions implemented. Psychological health education is typically provided only by nursing staff, lacking a comprehensive approach to addressing these emotional challenges.

The exclusion of patients older than 64 was primarily to avoid the significant confounding variables inherent in the geriatric population. Elderly patients present fundamental differences in the pathophysiology, clinical characteristics, and prognosis of traumatic fractures. They are commonly affected by osteoporosis, multiple comorbidities, and different injury mechanisms (predominantly low-energy falls), which collectively act as powerful confounders. Treating the elderly as a distinct population for research purposes is a widely accepted practice to ensure study precision. A recent scoping review highlighted that the management and outcomes of fractures in elderly patients should be considered a separate issue, owing to their unique profiles of osteoporosis, fall risk, and comorbidities (15). Furthermore, extensive evidence identifies age as a strong, independent risk factor for post-traumatic outcomes. Even with comparable injury severity scores, elderly trauma patients exhibit significantly higher mortality rates than their younger counterparts. A landmark study demonstrated that the post-trauma mortality rate in elderly patients was double that of younger adults, with a higher incidence of delayed mortality attributed to pre-injury comorbidities and a greater propensity for post-injury complications (16). The inclusion of elderly patients would, therefore, have significantly skewed our analysis of the outcomes for the 18–64 age group, which represents the primary working-age population.

The mutual risk factors between perioperative pain and anxiety or depression have long been established (14). Depression and anxiety can exacerbate the perception of pain, while emotional disturbances are also a consequence of acute and chronic pain. When pain is alleviated, the accompanying depressive symptoms tend to gradually subside (17). Esketamine, a novel derivative of ketamine, exhibits superior analgesic and antidepressant properties compared to ketamine. It was initially approved by the U.S. Food and Drug Administration (FDA) on March 4, 2019, for the treatment of refractory depression. Singh et al. (18) conducted a double-blind, randomized controlled trial on patients with refractory depression, demonstrating that intravenous administration of esketamine at 0.2 or 0.4 mg/kg produced rapid and effective antidepressant effects within 40 min. The lower-dose group showed better antidepressant efficacy. Previous studies have also confirmed that intravenously administration of esketamine provides a rapid and significant antidepressant effect with good tolerability. The efficacy rates were reported as 50.0% at 24 h, 48.2% at 3 days, and 43.7% at 7 days (19). Currently, no recommended safe dosage range exists for esketamine in postoperative analgesia. Research on its antidepressant effects primarily focuses on postpartum depression, with limited reports involving other surgical patient populations. A review of related literature indicates that a continuous postoperative infusion of esketamine at doses of 0.02–0.03 mg/kg/h effectively achieves both analgesic and antidepressant effects (20). Based on this, we selected doses of 0.5 mg/kg and 0.75 mg/kg for the 24-h esketamine pump to evaluate and compare their efficacy in this study.

The antidepressant effects of esketamine are likely associated with its multiple pharmacological mechanisms (21). The prevailing hypothesis is that esketamine persistently blocks the action of glutamate on NMDA receptors while increasing glutamate concentrations in the synaptic cleft. This enhances the activation of α-amino-3-hydroxy-5-methyl-4-isoxazole-propionate (AMPA) receptors, which are involved in synaptic plasticity, neuronal development, and functional regulation. The enhanced AMPA receptor activation promotes the upregulation of BDNF expression, leading to increased protein synthesis and dendritic growth, thereby exerting its antidepressant effects (22). Emerging evidence also suggests that specific subjective experiences during esketamine treatment, such as transient dissociative phenomena, may contribute to its therapeutic effect by disrupting maladaptive cognitive-affective patterns (23). In this study, compared to the control group (C), both the low-dose group (L) and the high-dose group (H) showed significantly reduced HAMD scores on postoperative days 1 and 3. Compared to the L group, the H group exhibited a significant difference only on postoperative day 3. These findings indicate that continuous postoperative esketamine infusion effectively alleviates postoperative depression, with the high-dose group showing more pronounced effects on day 3. Notably, recent real-world evidence indicates that esketamine's antidepressant efficacy extends to core depressive features such as anhedonia, with effects that can be distinct from general symptom improvement (24). Additionally, our study demonstrated that compared to group C, both groups L and H exhibited significantly elevated BDNF levels at 24 and 48 h postoperatively. Compared to group L, group H showed a significantly greater increase in BDNF at 24 h postoperatively, highlighting a dose-dependent effect of esketamine on BDNF elevation. BDNF has been proven to play a critical role in the molecular mechanisms of depression (25). Evidence suggests that esketamine exerts its antidepressant effects by blocking NMDA receptors and increasing BDNF levels in the medial prefrontal cortex and hippocampus. Moreover, studies have indicated that plasma BDNF levels may reflect central BDNF levels, making it a potential biomarker for depression (26). Existing evidence from animal studies suggests a link between postoperative declines in BDNF levels and depressive symptoms (27), although further research is needed to establish this relationship definitively.

Our study revealed that both the H group and L group had significantly lower postoperative sufentanil consumption and PCA activation counts compared to the C group, with a statistically significant difference in PCA counts between the H and L groups. This suggests that postoperative esketamine infusion enhances analgesia, thereby significantly reducing the need for sufentanil. This reduction in opioid use might explain the lower incidence of postoperative nausea and vomiting observed in the H and L groups compared to the C group, which is consistent with previous research findings (28). Additionally, the incidence of nightmares was slightly higher in the H and L groups than in the C group, but this difference was not statistically significant. This favorable safety profile, with manageable side effects, is consistent with patient-reported experiences in larger clinical cohorts, which support the overall acceptability of esketamine treatment (29). No statistically significant differences were observed in other postoperative analgesia-related adverse reactions among the three groups. The comparison of Ramsay sedation scor**es** among the three groups showed no statistically significant differences, indicating that continuous esketamine infusion does not increase the risk of postoperative over-sedation. The known mechanism of esketamine combined with opioids for analgesia involves low-dose esketamine attenuating the overactivation of the ascending nociceptive pathways and reducing the release of pronociceptive substances such as dynorphins in the spinal cord. This results in reduced pain sensitization and enhanced pain tolerance (30).

Regarding postoperative pain relief, a key outcome measured by VAS scores, our results demonstrated that while the overall Group × Time interaction was not significant, the high-dose esketamine group (H) exhibited a statistically significant reduction in VAS scores compared to the control group specifically at the 12-h postoperative time point (T3). This transient but significant analgesic effect, occurring during a period of typically intense early postoperative pain, is clinically meaningful. The finding that significant VAS reduction was localized to a single time point, whereas the reduction in opioid consumption and PCA demands was sustained and robust across the entire 48-h postoperative period, suggests that esketamine's primary analgesic contribution in this setting may not be through providing profound direct analgesia, but rather through its ability to prevent central sensitization (31) and reduce opioid-induced hyperalgesia (32). This mechanism effectively “unmasks” the analgesic efficacy of background opioids and reduces the amount of opioid required to achieve patient comfort, a concept supported by the significantly lower PCA compressions. Therefore, the most salient finding for clinicians regarding pain relief is not a dramatic reduction in reported VAS scores, but the significant opioid-sparing effect achieved with esketamine co-administration (33), which translates directly into a reduced burden of opioid-related side effects, as evidenced by the lower incidence of nausea and vomiting (33, 34).

Surgery, as a form of trauma treatment, is also a major source of postoperative stress. Inflammatory factors undergo significant changes under surgical stress, with IL-6 often serving as a stress indicator of pain. IL-6 has also been shown to play an important role in the diagnosis and treatment of depression, as it is believed to trigger the body's immune response under stress (35). Haapakoski et al. (36) found that patients with depressive symptoms generally have elevated levels of IL-6, further supporting its role as a biomarker linking inflammation, stress, and depression. Esketamine, the S (+) enantiomer of ketamine, has also been confirmed to possess neuroprotective effects, including reducing neuroinflammation, promoting hippocampal neuronal plasticity, and inhibiting circulating branched-chain amino acid levels (37). Our study found that esketamine significantly reduced IL-6 levels within 24 h postoperatively, with more pronounced effects observed in the high-dose group. This indicates that esketamine effectively mitigates postoperative inflammatory responses, contributing to its therapeutic benefits.

This study has several limitations that should be considered when interpreting the results. First, it was conducted at a single center, which may limit the generalizability of the findings. Second, the follow-up period was relatively short (7 days for the primary psychological outcome), preventing assessment of the long-term sustainability of esketamine's antidepressant and analgesic effects, as well as the evaluation of potential long-term adverse effects. Third, the assessment of depressive status relied solely on the HAMD scale. While it is a standardized tool, the lack of more comprehensive psychiatric evaluation or objective biomarkers for depression remains a limitation. Fourth, although the groups were well-balanced in measured baseline and surgical characteristics, the influence of unmeasured confounding factors (e.g., socioeconomic status, individual pain tolerance, level of social support) cannot be entirely ruled out. Finally, as noted, the selected subjects were restricted to patients with lower limb fractures. Whether esketamine exhibits similar antidepressant effects and the appropriate dosage for other types of traumatic fractures remains to be further investigated in future research. Future large-scale, multicenter randomized controlled trials with longer follow-up durations, incorporating more comprehensive psychological assessment tools, and encompassing diverse traumatic fracture populations are warranted to confirm and extend our findings. Furthermore, the application of advanced predictive analytics, such as machine learning models trained on multimodal data, holds promise for identifying which patients are most likely to benefit from esketamine therapy, paving the way for personalized treatment approaches in perioperative medicine (38).

In summary, continuous infusion of esketamine at doses of 0.5 mg/kg to 0.75 mg/kg for postoperative analgesia in patients with traumatic lower limb fractures effectively reduces postoperative depression scores, with 0.75 mg/kg showing superior effects. This approach achieves comparable analgesic outcomes while significantly reducing opioid consumption, thereby lowering the incidence of adverse reactions such as nausea and vomiting. Additionally, esketamine infusion increases BDNF levels, decreases IL-6 levels, and optimizes trauma orthopedic anesthesia and multimodal postoperative analgesia. These effects collectively accelerate patient recovery and improve postoperative outcomes.

## Data Availability

The original contributions presented in the study are included in the article/supplementary material, further inquiries can be directed to the corresponding author.
